# Differentiating primary bone marrow failure syndromes in children: a retrospective analysis of early clinical and laboratory features

**DOI:** 10.3389/fped.2025.1557212

**Published:** 2025-06-05

**Authors:** Yan-Li Leng, Yu-Qi Zhao, Hong-Juan Li, Yan Gu, Yan Han, GuYu Ding, Xiaoyue Zhang, Xu Li, Hui-Di Feng, ZiYun Lin, Xiao-Wei Zhao, Hong-Mei Wang

**Affiliations:** ^1^ Department of Pediatrics, The First Affiliated Hospital of Shandong First Medical University & Shandong Provincial Qianfoshan Hospital, Jinan, Shandong, China; ^2^ Department of Pediatrics, Shandong Provincial Qianfoshan Hospital, Jining Medical University, Jinan, Shandong, China

**Keywords:** bone marrow failure syndrome, aplastic anemia, refractory cytopenia in children, idiopathic cytopenia of undetermined significance, early clinical characteristics

## Abstract

**Objective:**

To investigate the early clinical characteristics of different subtypes of primary bone marrow failure syndrome (BMFS) in children and identify predictive factors for early diagnosis, thereby improving the ability to differentiate BMFS at an early stage.

**Methods:**

A retrospective analysis was conducted on the clinical and laboratory data of children with primary BMFS who received regular follow-up visits exceeding one year at the First Affiliated Hospital of Shandong First Medical University from January 2020 to September 2024. Based on long-term follow-up results, the children were reclassified into subgroups, and early clinical features, blood counts, and bone marrow examination results were compared across groups. Univariate and multivariate analyses were performed.

**Results:**

A total of 167 pediatric patients with primary BMFS were included in this study, of whom 112 (67.1%) were diagnosed with aplastic anemia (AA), 34 (20.3%) with refractory cytopenia (RCC), and 21 (12.6%) with idoipathic cytopenia of undetermined significance (ICUS). Significant statistical differences were observed among the three groups in terms of gender, red blood cell and platelet transfusion volumes within the first three months of disease onset, infection incidence, initial platelet and neutrophil counts, the lowest platelet and neutrophil values during the early stage of the disease, initial reticulocyte (RET) count and percentage, mean corpuscular volume (MCV), mean corpuscular hemoglobin (MCH), mean corpuscular hemoglobin concentration (MCHC), red cell distribution width (RDW), bone marrow cellularity, number of megakaryocytes, enzyme-linked tissue staining for megakaryocytes, and dysplasia in bone marrow smears (*p* < 0.05). Among these, gender, initial RET count, and bone marrow cellularity were identified as independent predictors for AA (*p* < 0.01).

**Conclusion:**

Early manifestations of pediatric BMFS are characterized by pancytopenia and bone marrow hematopoietic failure; however, different subtypes exhibit variations in early clinical features and laboratory findings. Early identification of these characteristics may improve diagnostic accuracy and facilitate more effective clinical management.

## Introduction

Bone marrow failure syndrome (BMFS) is a heterogeneous group of disorders caused by damage to hematopoietic stem and/or progenitor cells, encompassing both inherited and acquired forms (1, 2) Acquired BMFS can be further classified into primary and secondary types (2–4). The etiology of primary BMFS remains unclear, with common disorders including aplastic anemia (AA), myelodysplastic syndrome (MDS), and idoipathic cytopenia of undetermined significance (ICUS). Although these conditions have distinct pathophysiological mechanisms (5–9), they share similar early clinical manifestations, peripheral blood counts, and bone marrow examination findings, making early diagnosis and differentiation particularly challenging. Accurately identifying primary BMFS in its early stages based on clinical features is crucial for timely and targeted treatment, ultimately improving therapeutic outcomes. However, this remains a significant challenge in clinical practice.

Among pediatric MDS subtypes, refractory cytopenia (RCC) is the most prevalent (10) and exhibits clinical similarities to AA. It is typically characterized by minimal or absent blasts and lacks specific cytogenetic and immunologic markers. In recent years, several studies have explored the differential diagnosis and prognostic distinctions between AA and RCC (8, 9), MDS and ICUS (7, 10, 11), as well as non-severe aplastic anemia (NSAA) and RCC (5, 9). However, most of these studies have been limited by small sample sizes, and comprehensive comparative analyses of the early clinical characteristics of AA, RCC, and ICUS remain scarce.

This study retrospectively analyzed the early clinical features of pediatric AA, RCC, and ICUS diagnosed at a single center. By examining differences in early disease presentation and identifying predictive diagnostic factors, we aim to provide scientific evidence to facilitate early recognition, enable stratified treatment, and ultimately improve prognosis in affected children.

## Materials and methods

### Patient selection

This study included pediatric patients diagnosed with primary BMFS who underwent regular follow-up for more than one year at the Department of Pediatric Hematology, First Affiliated Hospital of Shandong First Medical University, between January 2020 and September 2024. The inclusion criteria were as follows: (1) cytopenia[(white blood cell (WBC) count ≤4.0 × 10^9^/L; Neutrophil count ≤1.5 × 10^9^/L; Hemoglobin (Hb) levels by age: for 0–6 months, Hb < 100 g/L; for 6 months to 6 years, Hb < 110 g/L; for 6–16 years, Hb < 120 g/L; Platelet (PLT) count <100 × 10^9^/L. Based on the referenced National Health Commission of China guidelines and foreign guidelines (12–18)] affecting one or more peripheral blood lineages. (2) Bone marrow biopsy showing age-adjusted normal or decreased cellularity (19), with reticulin fibrosis grade ≤1; (3) no detection of PNH clones and negative for PNH-related gene mutations; (4) initial diagnosis before 18 years of age; (5) informed consent obtained from the patient and their legal guardians; (6) exclusion of inherited BMFS[This was based on: “a combination of detailed clinical evaluation for characteristic physical findings, family history review, and, where clinically indicated, specific investigations such as chromosomal breakage studies for Fanconi Anemia or targeted genetic testing for common inherited BMFS syndromes using (e.g., next-generation sequencing panels) or cytogenetic studies including fluorescence *in situ* hybridisation (FISH).” Patients with confirmed or highly suspected inherited BMFS based on this workup were excluded.], myeloproliferative neoplasm-associated bone marrow fibrosis, leukemia, lymphoma, non-hematologic malignancies, transient erythroblastopenia of childhood, and metabolic disorders that could contribute to bone marrow failure; (7) exclusion of patients with a history of prior chemotherapy, radoitherapy, or exposure to bone marrow-toxic medications.

### Collection of clinical and laboratory data

The following clinical and laboratory parameters were collected: (1) general characteristics, including age at initial diagnosis and sex; (2) clinical symptoms and signs, including initial clinical presentation, incidence of infections within three months of disease onset, bleeding status, and transfusion history; (3) peripheral blood counts and RET parameters, including absolute values of the three major hematopoietic lineages in the complete blood count at disease onset, lowest values of these lineages within the first three months after onset, and initial RET count and percentage; (4) red blood cell indices, including mean corpuscular volume (MCV), mean corpuscular hemoglobin (MCH), mean corpuscular hemoglobin concentration (MCHC), and red cell distribution width (RDW), measured at disease onset; (5) bone marrow examination, including the degree of bone marrow cellularity in the initial bone marrow smear, erythroid and granulocytic proliferation status, megakaryocyte count, bone marrow biopsy findings, morphological abnormalities of bone marrow cells, and detection of micromegakaryocytes via enzyme labeling.

### Diagnostic and classification criteria

The diagnosis and classification of pediatric AA were based on the Diagnosis and Treatment Guidelines for Pediatric Aplastic Anemia (2019 edition) issued by the National Health Commission of China (20, 21). Specifically, there is persistent reduction in blood cell counts; bone marrow aspiration shows active or reduced proliferation of nucleated cells, decreased hematopoietic cells in marrow fragments [Guidelines from the Marrow Failure Study Group of the Pediatric Haemato-Oncology Italian Association (AIEOP): hematopoietic cells decrease to below 30%, and fat cells replace the hematopoietic cells (22)], and increased ratios of non-hematopoietic cells (such as lymphocytes, reticular cells, plasma cells, mast cells, etc.); a significant reduction or absence of megakaryocytes, and a noticeable decrease in erythroid and myeloid lineages; bone marrow biopsy reveals reduced proliferation of nucleated cells, decreased or absent megakaryocytes, reduced hematopoietic tissue, increased fat and/or non-hematopoietic cells, no fibrous tissue hyperplasia, and no abnormal cell infiltration; congenital and other acquired or secondary bone marrow failure syndromes must be excluded.

The diagnosis criteria for aplastic anemia (AA) classification (20, 21):Severe aplastic anemia (SAA): (1) Bone marrow nucleated cell proliferation is between 25% and 50%, with residual hematopoietic cells less than 30%, or nucleated cell proliferation is below 25%; (2) Peripheral blood criteria must meet at least 2 of the following 3 items: ① Absolute neutrophil count <0.5 × 10^9^/L; ② Platelet count <20 × 10^9^/L; ③ Absolute reticulocyte count <20 × 10^9^/L, or corrected reticulocyte percentage <1%.Very severe aplastic anemia (VSAA): In addition to meeting the criteria for SAA, absolute neutrophil count <0.2 × 10^9^/L. Non-severe aplastic anemia (NSAA): Does not meet the diagnostic criteria for SAA and VSAA.

The diagnosis and classification of RCC were based on established international and domestic criteria (8, 10): (1) Peripheral blood cell decrease: One or more lineages of blood cells show varying degrees of persistent reduction for more than 3 months with an unknown cause. (2) Dysplastic hematopoietic cell morphology: Bone marrow smears and biopsies show dysplasia in two or more lineages of bone marrow cells, or dysplastic morphology and development abnormalities in one lineage of bone marrow cells, with the proportion of dysplastic cells greater than 10%. (3) Blasts: Bone marrow blast percentage <5%, peripheral blood blast percentage <2%, excluding other types of myelodysplastic syndromes (MDS). (4) Cytogenetic abnormalities: Bone marrow cells exhibit genetic abnormalities and clonal chromosomal karyotype abnormalities, but must exclude cytogenetics related to acute myeloid leukemia (AML). (5) Exclusion of other hematological or non-hematological diseases that could cause cytopenia and dysplastic hematopoietic cell development abnormalities.

The diagnostic criteria (23, 24) for ICUS include persistent (≥6 months) reduction of one or more blood cell lineages; failure to meet the minimum diagnostic criteria for MDS; and exclusion of other hematologic or non-hematologic diseases. To achieve this diagnosis of exclusion, evaluations typically included repeated complete blood counts, bone marrow aspiration/biopsy review (including morphology and cytogenetics), PNH clone testing, viral studies, nutritional assessments, and screening for autoimmune diseases.

Pathological Hematopoiesis refers to abnormalities in the hematopoietic system that lead to the production of blood cells with functional or morphological defects. The proportion of morphologically abnormal cells must meet the criteria of affecting two or more lineages or one lineage exceeding 10%. The morphological characteristics of developmental abnormalities in each lineage include (8, 10):

Erythroid Lineage: Nuclear budding, nuclear bridging, nuclear fragmentation, increased nuclear lobation, megaloblastic changes, ringed sideroblasts, vacuolation, and positive glycogen staining, among others;
Granulocyte Lineage: Abnormal nuclear lobation (either excessive or insufficient), reduced cytoplasmic granules, pseudo-Chediak-Higashi granules, and Auer rods;
Megakaryocyte Lineage: Small megakaryocytes, absence of nuclear lobation, and multinucleated megakaryocytes, among other abnormalities.

In this study, the criteria for interpreting the morphological development abnornalities of bone marrow cells reference the relevant standards/guidelines for RCC. This term is conceptually equivalent to ineffective hematopoiesis or dysplastic hematopoiesis and is used interchangeably in the text.

The grading criteria for the degree of hematopoietic hyperplasia based on age-adjusted hematopoietic area (The normal reference range for the proportion of nucleated cells in bone marrow, adjusted for age, is as follows (19): for patients aged 20–30 years, it is 60% to 70%; for those aged 40–60 years, it is 40% to 50%; and for individuals aged 70 years and older, it is 30% to 40%) in bone marrow biopsies are as follows (25): (1) severely decreased hyperplasia: hematopoietic area <20%; (2) mildly decreased hyperplasia: 20%≤ hematopoietic area <40%; (3) approximately normal: 40%≤hematopoietic area <60%; (4) moderately active:60% ≤ hematopoietic area <80%; (5) extremely active: hematopoietic area ≥80%^.^

All cases were comprehensively diagnosed based on clinical presentation, peripheral blood counts, bone marrow smears, bone marrow biopsies, cytogenetic and genetic analyses, and long-term follow-up data. Diagnoses were independently reviewed multiple times by two experts. In cases of disagreement, further discussion was conducted to reach a consensus.

### Follow-up

Follow-up was conducted until September 30, 2024. Clinical data were collected at initial diagnosis, as well as at +3 months, +6 months, +12 months, and +2 years. Follow-up information was obtained from hospitalization records, outpatient medical records, and telephone interviews. For patients who died during the follow-up period, death was confirmed through medical records or direct communication with family members.

### Statistical analysis

Data analysis was performed using SPSS 26.0 software. Continuous variables with a normal distribution were expressed as mean ± SD, while non-normally distributed data were presented as median (range). Categorical variables were reported as frequencies or percentages. For group comparisons, one-way ANOVA was used for normally distributed continuous variables, while the Kruskal–Wallis *H* test was applied for non-normally distributed data. Categorical variables were compared using the chi-square test (*χ*^2^ test) or Fisher's exact test, as appropriate. Variables that were statistically significant in univariate analysis were included in a multivariate logistic regression analysis to identify independent predictors. A *P*-value <0.05 was considered statistically significant.

## Results

### Baseline characteristics

**Figure 1:**
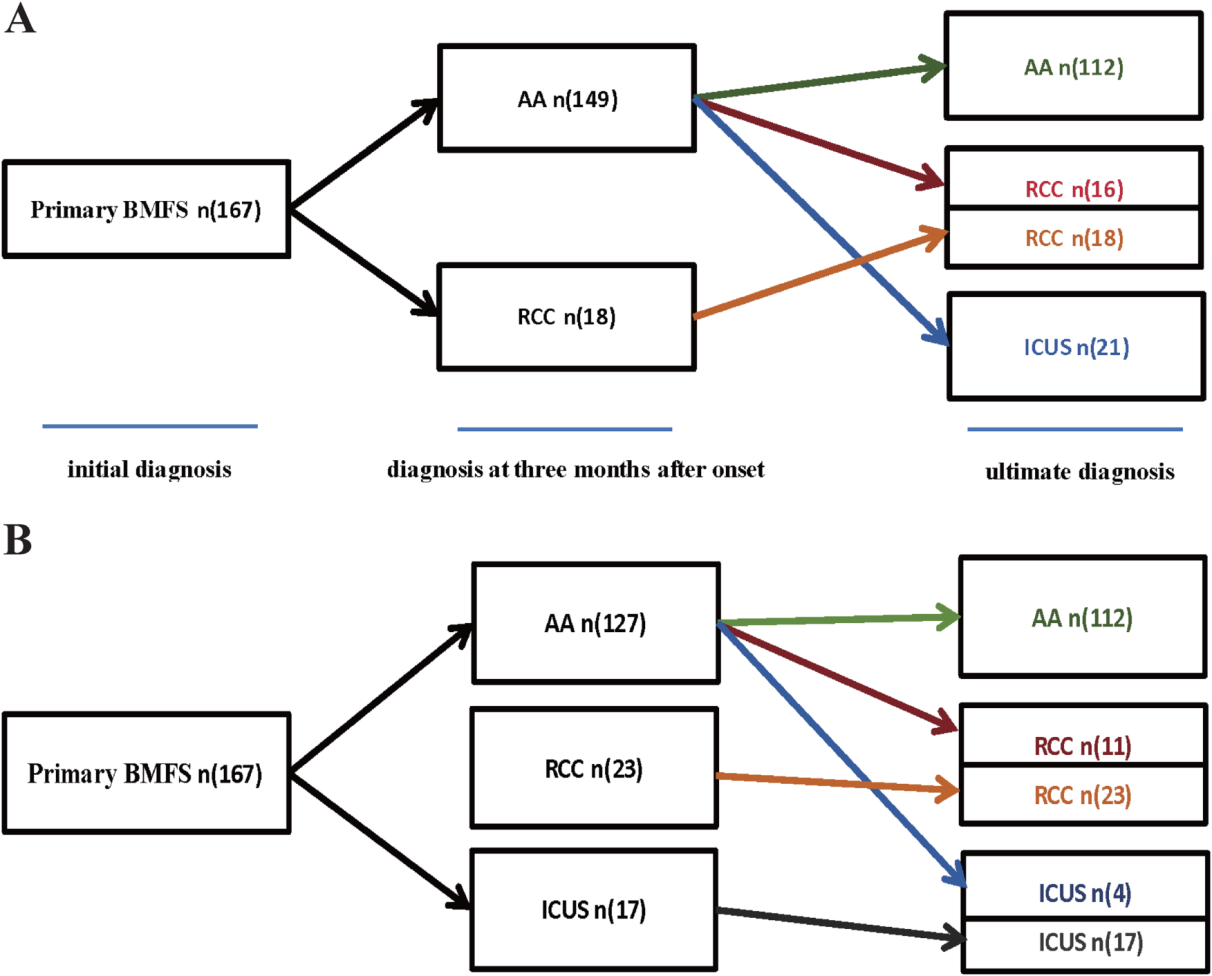
**(A)** The diagnoses of 167 children with Primary BMFS. **(A)** Among 167 cases of Primary BMFS, initially, 149 cases were AA and 18 were RCC, ultimately,112 (67.07%) were classified as AA, 34 (20.36%) as RCC, and 21 (12.57%) as ICUS. 37 patients (24.83%) were reclassified during follow-up,16 children were rediagnosed as RCC, and 21 children were rediagnosed as ICUS. **(B)** We used independent predictive factors (such as initial RET count, sex, and degree of bone marrow hyperplasia) obtained from the multifactorial analysis in this study to participate in the diagnosis of primary BMFS, initially, 127 cases were AA, 23 were RCC and 17 were ICUS, ultimately,112 (67.07%) were classified as AA, 34 (20.36%) as RCC, and 21 (12.57%) as ICUS. 15 patients (11.81%) were reclassified during follow-up,11 children were rediagnosed as RCC, and 4 children were rediagnosed as ICUS.

Our study initially included 167 children diagnosed with primary Bone Marrow Failure Syndromes (BMFS), with a median age of 9 years (range: 1–18 years). Among them, 97 (58.1%) were male and 70 (41.9%) were female. Based on their initial diagnosis, they primarily classified as AA (*n* = 149) or RCC (*n* = 18), as shown in Figure 1. After more than one year of follow-up, a comprehensive re-evaluation was conducted based on clinical course evolution, repeat laboratory tests, bone marrow examinations (including morphology, pathology, and enzyme-linked staining), as well as genetic/cytogenetic analyses.

**Figure 2:**
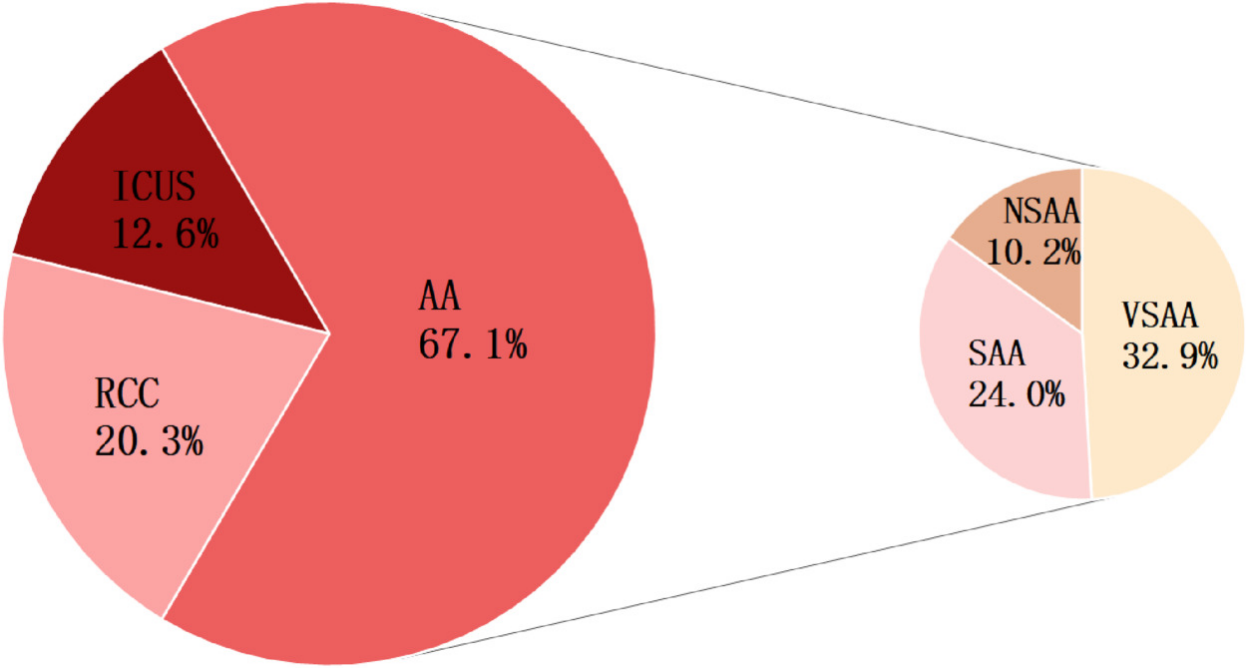
Final diagnoses of 167 children with Primary BMFS. Among 167 cases of Primary BMFS,112 (67.07%) were classified as AA, 34 (20.36%) as RCC, and 21 (12.57%) as ICUS. there were 55 (32.94%) VSAA, 40 (23.95%) SAA, and 17 (10.18%) NSAA. The figure displays the percentage representation of each group.

Ultimately, the diagnoses of 167 patients who completed follow-up were reclassified into three groups: 112 cases (67.1%) were diagnosed with AA, 34 (20.3%) with RCC, and 21 (12.6%) with ICUS. Within the AA group, 55 patients (49.1%) were classified as VSAA, 40 (35.7%) as SAA, and 17 (15.2%) as NSAA, as shown in Figure 2.

Among the 149 patients initially diagnosed with AA, 37 patients (24.83%) were reclassified during follow-up. Specifically, 16 children were rediagnosed as RCC, and 21 children were rediagnosed as ICUS. The RCC group increased from the initial diagnosis of 18 cases to a final diagnosis of 34 cases. The ICUS group (*n* = 21) was entirely composed of patients initially diagnosed with AA, as shown in Figure 1A.

**Figure 3:**
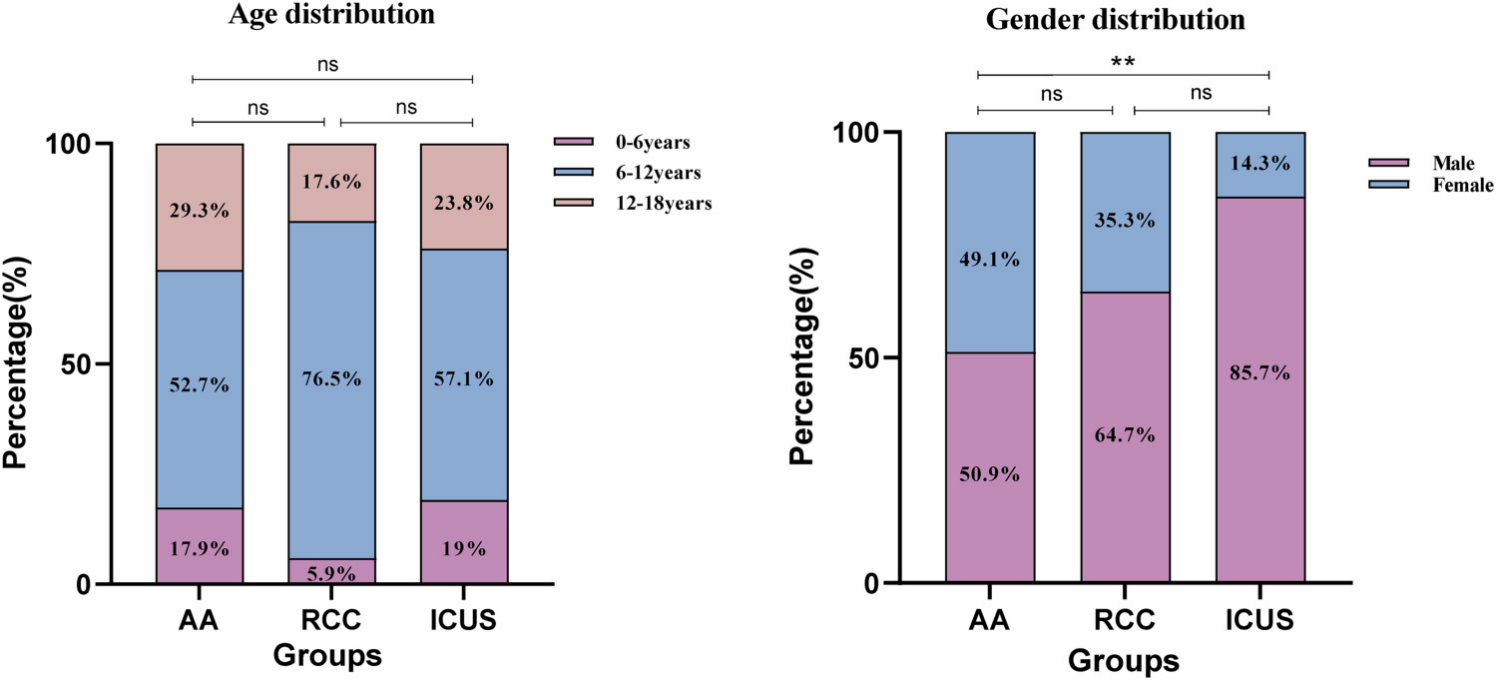
Gender and age distribution of the three groups of children. **(A)** The distribution of the three groups across the age ranges of 0–6 years, 6–12 years, and 12–18 years. **(B)** The distribution of the three groups across gender. Data are presented as frequency (percentage %). *P ≤ 0.05, **P ≤ 0.01, ***P ≤ 0.001, ns *P* > 0.05. Comparison among the three groups (χ² test or Fisher's exact test).

There were no significant differences in the age of onset among the three groups, with the majority of patients diagnosed between 6 and 12 years of age (Figure 3). Males were predominant in all groups; however, the proportion of male patients in the ICUS group (85.7%) was significantly higher than that in the AA group (50.9%) (*χ*^2^ = 8.7, *P* = 0.003).Detailed data are presented in Table 1.

**Table 1:** Baseline characteristics and Clinical Features of Pediatric Patients with Primary BMFS.

Variable	Tobal (*n* = 167)	AA (*n* = 112)	RCC (*n* = 34)	ICUS (*n* = 21)	*H*/*X*^2^-value	*P*-value
Age (years)	9 (1–18)	9 (1–18)	9 (4–17)	8 (2–15)	0.8	0.684
0–6	26 (15.6)	20 (17.9)	2 (5.9)	4 (19)	4.4	0.363
6–12	97 (58.1)	59 (52.7)	26 (76.5)	12 (57.1)		
12–18	54 (32.3)	33 (29.4)	13 (17.6)	8 (23.8)		
Gender					11.3	0.002
Male	97 (58.1)	57 (50.9)	22 (64.7)	18 (85.7)		
Female	70 (41.9)	55 (49.1)	12 (35.3)	3 (14.3)		
Initial Clinical Presentation					10.6	0.515
Bleeding	65 (38.9)	40 (35.7)	13 (38.2)	12 (57.1)		
Fever	20 (12)	16 (14.3)	2 (5.9)	2 (9.5)		
Pallor and fatigue	15 (9)	7 (6.3)	6 (17.6)	2 (9.5)		
Bleeding and Fever	35 (21)	27 (24.1)	5 (14.7)	3 (14.3)		
Bleeding and Pallor and fatigue	17 (10.2)	11 (9.8)	5 (14.7)	1 (4.8)		
Fever and Pallor and fatigue	7 (4.2)	5 (4.5)	1 (2.9)	1 (4.8)		
Fever and Bleeding and Pallor and fatigue	8 (4.8)	6 (5.4)	2 (5.9)	0 (0)		
Volume of red blood cell transfusion (one U)	3 (0–17)	4 (0–16)	1 (0–8)	0 (0–17)	14.2	0.001
Volume of platelet transfusion (one therapeutic dose)	3 (0–28)	3 (0–19)	1 (0–6)	1 (0–28)	20.5	0.000
Bleeding frequency within 3M of onset	2 (0–7)	2 (1–7)	1 (0–4)	2 (0–5)	5.2	0.074
Infection frequency within 3M of onset	2(0–8)	2(0–8)	1(0–4)	1(0–4)	10.4	0.006

Note: Continuous data are expressed as median (range), while categorical data are presented as frequency (percentage%). The shown *p*-value derived from the comparison across three groups, the comparison of median ages among the three groups was conducted using the Kruskal–Wallis *H* test. The distribution of different age groups among the three patients and the comparison of initial clinical presentations were analyzed using the chi-square test (*χ*² test).

### Early clinical manifestations among the three groups

A retrospective analysis of the initial clinical manifestations revealed that bleeding was the most common presenting symptom across all groups. Isolated bleeding was the predominant feature in 40 patients (35.7%) in the AA group, 13 (38.2%) in the RCC group, and 12 (57.1%) in the ICUS group. The second most frequent presentation in the AA and ICUS groups was combined bleeding and fever (AA: 27 cases, 24.1%; ICUS: 3 cases, 14.3%), whereas in the RCC group, pallor and fatigue (6 cases, 17.6%) were more common. However, there were no statistically significant differences in symptom distribution among the three groups. Additionally, no significant differences were observed in the frequency of bleeding episodes within the first three months of disease onset.

During the early disease stage (within three months of onset), the infection rate in the AA group was significantly higher than that in the RCC group (*H* = 28.1, *P* = 0.006). Moreover, red blood cell and platelet transfusion volumes within the first three months were significantly higher in the AA group than in the RCC group (RBC: *H* = 31.0, *P* = 0.003; Platelets: *H* = 38.2, *P* < 0.001). Additionally, the platelet transfusion volume in the AA group was significantly higher than that in the ICUS group (*H* = 30.6, *P* = 0.021). These findings indicate that patients with AA are more likely to experience frequent infections and require transfusion support during the early stages of the disease. Detailed data are presented in Table 1.

### Early peripheral blood counts, reticulocyte levels and red blood cell indices among the three groups

**Figure 4:**
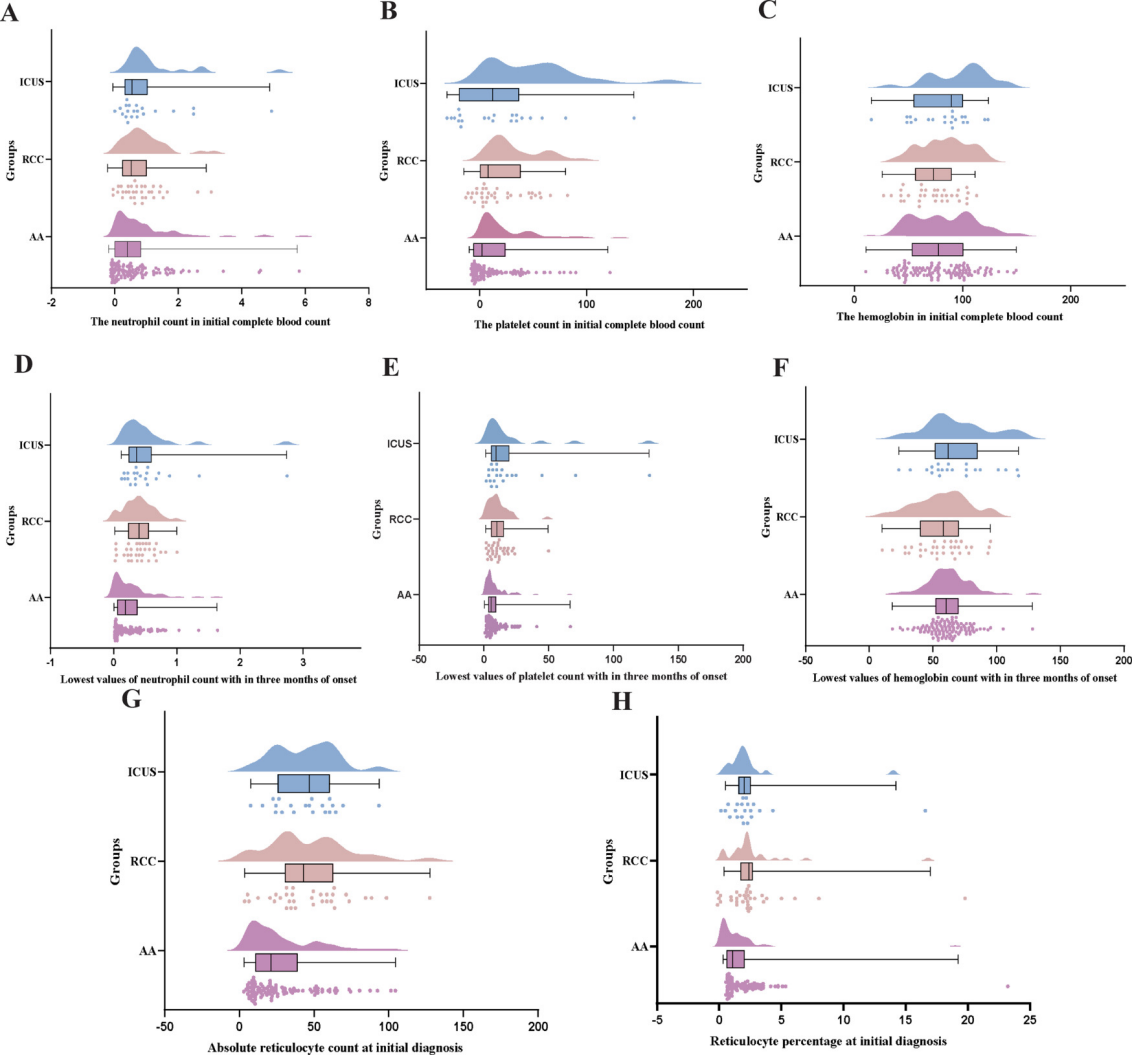
Comparison of early hematological parameters Among AA, RCC, and ICUS groups. **(A–C)** the comparison of the neutrophil count(×10 × 10^9^/L) and the hemoglobin (g/L) and the platelet count (×10 × 10^9^/L) in initial complete blood count among the three groups. **(D–F)** the comparison of the lowest values of neutrophil count (×10 × 10^9^/L) and the hemoglobin (g/L) and the platelet count (×10 × 10^9^/L) with in three months of onset among the three groups. **(G,H)** The comparison of absolute reticulocyte count (×10 × 10^9^/L)and reticulocyte percentage (%) at initial diagnosis among the three groups. Data are presented as median (range) (the Kruskal–Wallis *H* test).

Univariate analysis revealed significant differences in initial platelet (PLT) and absolute neutrophil count (ANC) levels among the AA, RCC, and ICUS groups (Figures 4A,C). Specifically, the initial PLT level in the AA group was significantly lower than that in the RCC group (*H* = −23.3, *P* = 0.041), whereas the ICUS group exhibited significantly higher initial PLT (*H* = −33.2, *P* = 0.012) and ANC levels (*H* = −26.9, *P* = 0.048) compared to the AA group.

During the first three months after disease onset, the lowest PLT and ANC values in the AA group were significantly lower than those in both the RCC (PLT: *H* = −23.9, *P* = 0.034; ANC: *H* = −32.3, *P* = 0.002) and ICUS groups (PLT: *H* = −29.4, *P* = 0.031; ANC: *H* = −38.6, *P* = 0.002), suggesting more severe hematopoietic impairment in the early disease stage in the AA group (Figures 4D,F).

Furthermore, reticulocyte (RET) analysis demonstrated that both the absolute RET count and RET percentage in the AA group were significantly lower than those in the RCC (absolute count: *H* = 34.3, *P* = 0.001; percentage: *H* = 38.1, *P* < 0.001) and ICUS groups (absolute count: *H* = 36.4, *P* = 0.004; percentage: *H* = 33.5, *P* = 0.009) (Figures 4G,H). These findings indirectly indicate that bone marrow failure was most severe in the AA group.

Univariate analysis revealed significant differences in MCV, MCH, and RDW among the AA, RCC and ICUS groups (MCV: *H* = 8.1, *P* = 0.018; MCH: *H* = 6.6, *P* = 0.037; RDW: *H* = 10.9, *P* = 0.004). Specifically, MCV (*H* = −23.6, *P* = 0.026) MCH (*H* = −22.4, *P* = 0.038) and RDW (*H* = −24.5, *P* = 0.018) were significantly lower in the AA group compared to the RCC group. However, no statistically significant differences were observed in these parameters between the AA and ICUS groups or between the RCC and ICUS groups (Table 2).

**Table 2:** Comparison of early-stage red blood cell parameters among the AA, RCC, and ICUS groups.

Variable	Tobal (*n* = 167)	AA (*n* = 112)	RCC (*n* = 34)	ICUS (*n* = 21)	*H*/*X*^2^-value	*P*-value
MCV (fL)	87.9 (75.3–126.2)	86.2 (75.3–126.2)	95.2 (83.1–112)	92.4 (84.6–110.6)	8.1	0.018
MCH (pg)	31.1 (19.1–39.1)	30.6 (19.1–39.1)	33.8 (29.4–39.0)	31.4 (30.1–36.3)	6.6	0.037
MCHC (g/L)	347.0 (283.0–391.0)	347.0 (283.0–391.0)	347.0 (321.0–364.0)	347.0 (327.0–356.0)	0.7	0.701
RDW (%)	13.8 (10.7–24.4)	13.5 (10.7–19.4)	15.0 (12.7–24.4)	15.2 (12.5–24.1)	10.9	0.004

Note: Continuous data are expressed as median (range), while categorical data are presented as frequency (percentage%). MCV, mean corpuscular volume, MCH, mean corpuscular hemoglobin, MCHC, mean corpuscular hemoglobin concentration, and RDW: red cell distribution width, measured at disease onset. The shown *p*-value derived from the comparison across three groups. The distribution of MCV, MCH, RDW among the three patients was conducted using the Kruskal–Wallis *H* test.

### Early bone marrow examination findings among the three groups

#### Bone marrow cellularity and megakaryocyte counts

Significant differences in bone marrow cellularity were observed among the three groups based on the initial bone marrow smears. The ICUS group (17 cases, 81%) and RCC group (24 cases, 70.6%) predominantly exhibited active bone marrow hyperplasia, whereas the AA group (86 cases, 76.8%) primarily showed bone marrow hypoplasia (*X*^2^ AA vs. RCC = 25.9, *P* < 0.001; *X*^2^ AA vs. ICUS = 26.9, *P* < 0.001).

**Figure 5:**
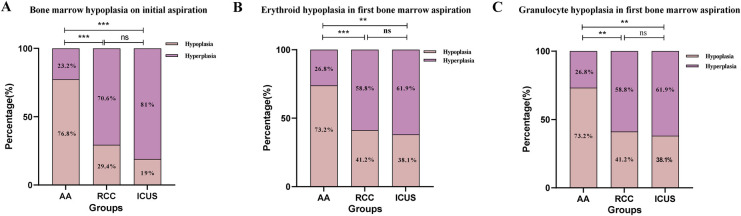
Comparison of bone marrow proliferation among the three groups based on initial bone marrow aspiration. **(A)** The comparison of the bone marrow proliferation on initial aspiration among the three groups. **(B)** The comparison of the erythroid hypoplasia deree in first bone marrow aspiration among the three groups. **(C)** The comparison of the granulocyte hypoplasia degree in first bone marrow aspiration among the three groups. Data are presented as frequency (percentage %). *P ≤ 0.05, **P ≤ 0.01, ***P ≤ 0.001, ns *P* > 0.05. Comparison among the three groups (χ² test or Fisher's exact test).

**Figure 6:**
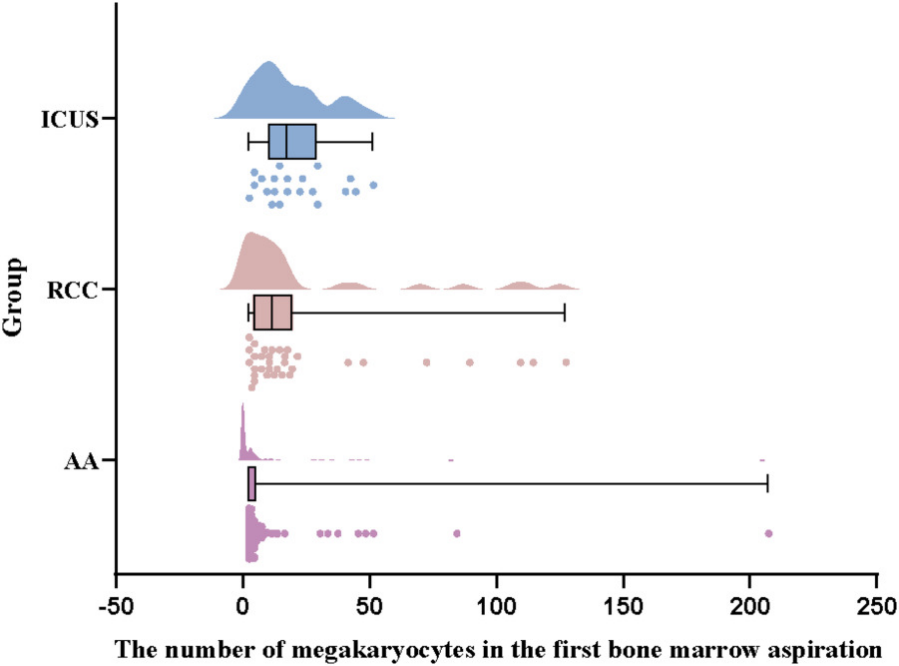
Comparison of megakaryocyte counts among the three groups**.** Data are presented as median (range) (the Kruskal–Wallis *H* test).

Further analysis of hematopoietic lineage proliferation demonstrated that erythroid proliferation was predominantly decreased in all three groups, with the AA group exhibiting the highest proportion of erythroid hypoplasia (82 cases, 73.2%), significantly greater than that in the ICUS group (13 cases, 61.9%) and RCC group (20 cases, 58.8%) (*X*^2^ AA vs. RCC = 26.0, *P* < 0.001; *X*^2^ AA vs. ICUS = 11.9, *P* = 0.001). Similarly, granulocytic proliferation was primarily reduced across all groups (AA group: 91 cases, 81.3%; RCC group: 19 cases, 55.9%; ICUS group: 11 cases, 52.4%). The AA group demonstrated a significantly higher proportion of granulocytic hypoplasia compared to the RCC group and ICUS group (*X*^2^ AA vs. RCC = 9.0, *P* = 0.004; *X*^2^ AA vs. ICUS = 8.2, *P* = 0.007), as illustrated in Figure 5. Regarding megakaryocyte counts, the RCC group and ICUS group had significantly higher megakaryocyte numbers than the AA group (*X*^2^ AA vs. RCC = −50.8, *P* < 0.001; *X*^2^ AA vs. ICUS = −61.7, *P* = 0.006), as shown in Figure 6.

**Figure 7:**
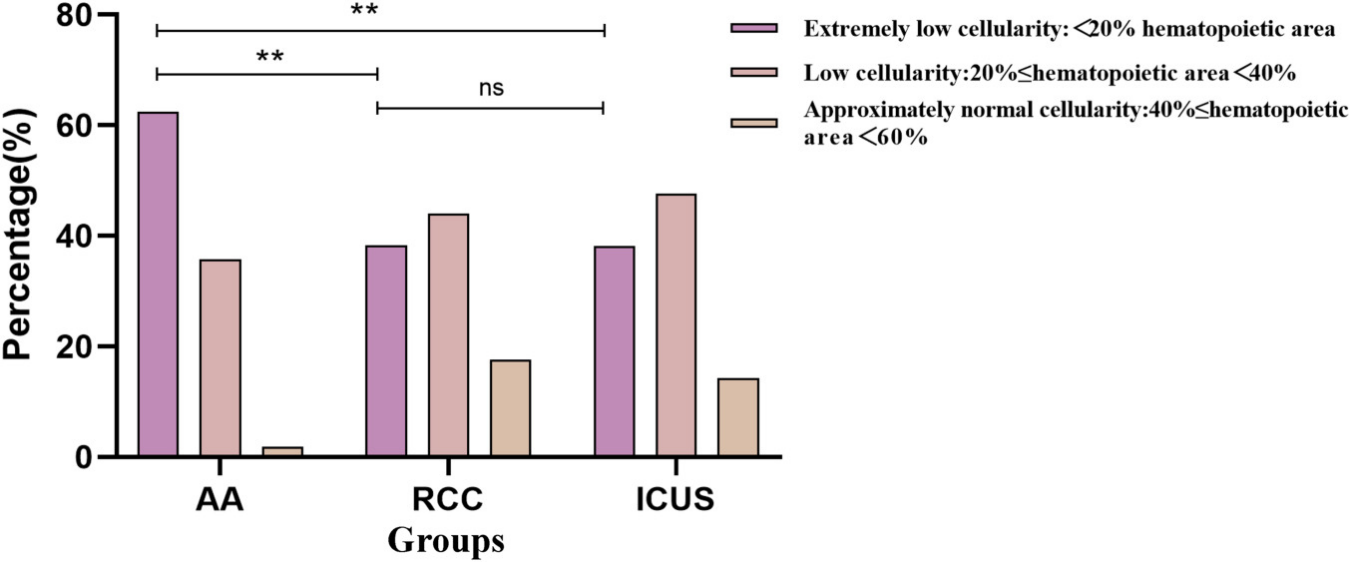
Comparison of bone marrow cellularity Among the three groups based on initial bone marrow biopsy. Data are presented as frequency (percentage%).*P ≤ 0.05, **P ≤ 0.01, ***P ≤ 0.001, ns *P* > 0.05. Comparison among the three groups (*χ*² test or Fisher's exact test).

Based on the pathological grading criteria for bone marrow hematopoietic area, bone marrow biopsy results were classified into Extremely low cellularity (<20% hematopoietic area), low cellularity (20%<40%), and approximately normal cellularity (40%−60%). Although all three groups primarily exhibited bone marrow hypoplasia, the distribution of cellularity levels differed significantly. The AA group had the highest proportion of cases with severely reduced cellularity (70 cases, 62.5%), which was significantly higher than in the RCC group (13 cases, 38.2%) and ICUS group (8 cases, 38.1%) (*X*^2^ AA vs. RCC = 10.4, *P* = 0.005; *X*^2^ AA vs. ICUS = 8.2, *P* = 0.012), as depicted in Figure 7.

#### Dysplasia in bone marrow

Bone marrow dysplasia, a requisite criterion for the diagnosis of MDS, showed significant differences in prevalence among the three groups. The RCC group exhibited a significantly higher proportion of dysplasia (100%) compared to the ICUS group (57.1%) and the AA group (25%) (*X*^2^_RCC vs. ICUS_ = 17.4, *P* < 0.001; *X*^2^_RCC vs. AA_ = 60.0, *P* < 0.001; *X*^2^_ICUS vs. AA_ = 8.7, *P* = 0.005).

Regarding the distribution of dysplasia, the AA group was predominantly characterized by no dysplasia (75%) or single-lineage dysplasia (23.2%). In contrast, the RCC group had a higher prevalence of single-lineage dysplasia (52.9%) or multilineage dysplasia (47.1%), while the ICUS group primarily exhibited no dysplasia (38.1%) or single-lineage dysplasia (57.1%).

**Figure 8:**
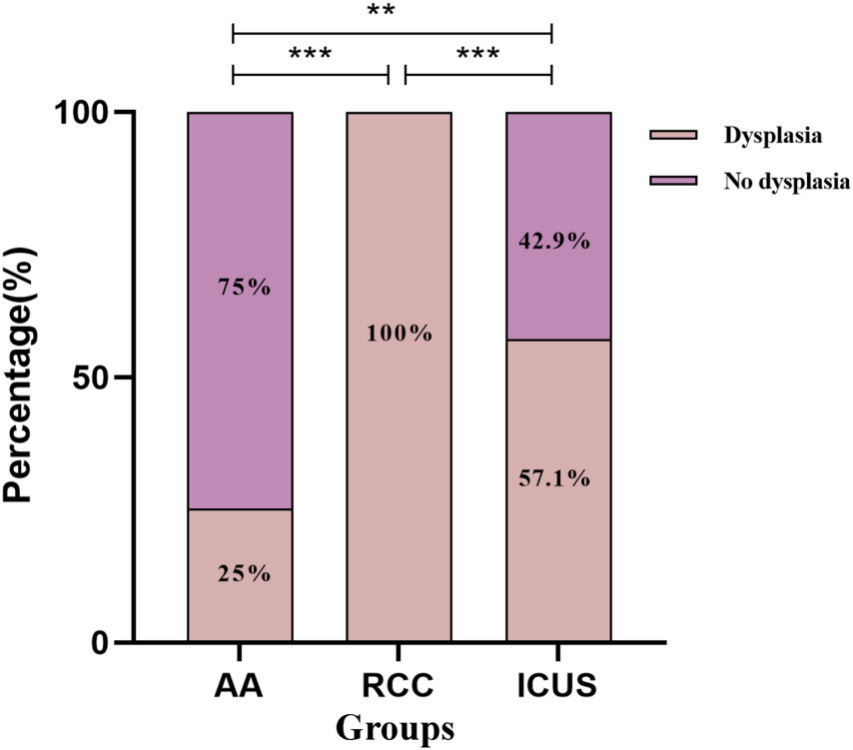
Comparison of bone marrow dysplasia among the three groups. Data are presented as frequency (percentage%). *P ≤ 0.05, **P ≤ 0.01, ***P ≤ 0.001, ns *P* > 0.05. Comparison among the three groups (*χ*² test or Fisher's exact test).

Comparative analysis revealed that the proportion of single-lineage and multilineage dysplasia in the RCC group was significantly higher than that in both the AA group and the ICUS group (*X*^2^_AA vs. RCC_ = 76.5, *P* < 0.001; *X*^2^_AA vs. ICUS_ = 11.1, *P* = 0.004; *X*^2^_RCC vs. ICUS_ = 20.5, *P* < 0.001). Detailed data are presented in Figure 8.

#### Dysplastic megakaryocyte characteristics

Among the 167 patients with primary BMFS, 111 underwent enzyme-linked tissue staining for megakaryocytes, revealing dysplastic megakaryocytes in 45 cases (40.5%). The prevalence and morphological classification of dysplastic megakaryocytes varied significantly among the three groups, with the highest frequency observed in the RCC group (31 cases, 27.9%), which was significantly higher than in the AA group (8 cases, 7.2%) and the ICUS group (6 cases, 5.4%).

In the AA group, the predominant dysplastic megakaryocyte forms were large mononucleated small megakaryocytes (6 cases, 75%) and binucleated megakaryocytes (3 cases, 37.5%), with occasional multinucleated megakaryocytes (1 case, 12.5%).In contrast, the RCC group exhibited a higher prevalence of megakaryocytic dysplasia, with large mononucleated small megakaryocytes being the most common (24 cases, 77.4%), followed by binucleated megakaryocytes (18 cases, 58.1%), multinucleated megakaryocytes (12 cases, 38.7%), mononucleated small megakaryocytes (6 cases, 19.4%), and binucleated small megakaryocytes (5 cases, 16.1%).The ICUS group demonstrated a lower prevalence of megakaryocytic dysplasia, primarily characterized by multinucleated megakaryocytes (3 cases, 50.0%) and large mononucleated small megakaryocytes (3 cases, 50%), with a smaller proportion of binucleated megakaryocytes (1 case, 16.7%) and mononucleated small megakaryocytes (1 case, 16.7%).

These findings indicate that dysplastic megakaryocyte characteristics differ among BMFS subtypes, with RCC exhibiting the highest prevalence and the most diverse dysplastic forms.

### Independent predictors of AA

**Figure 9:**
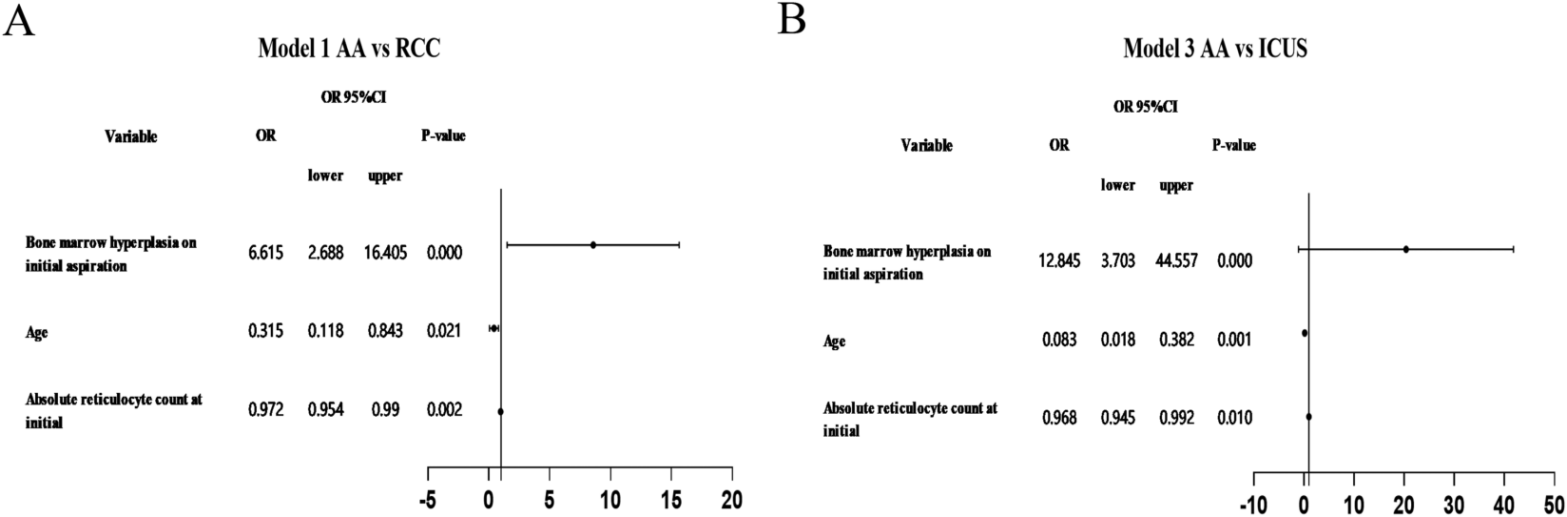
Forest plot of multivariate logistic regression analysis. **(A)** Using RCC as the control in logit model 1 for comparing AA with RCC **(B)** Using ICUS as the control in logit model 1 for comparing AA with ICUS.

Multivariate logistic regression analysis identified independent predictors distinguishing AA from ICUS, including initial reticulocyte (RET) count (*P* = 0.010), sex (*P* = 0.001), and bone marrow cellularity (*P* < 0.001). Similarly, when comparing AA with RCC, the independent predictors were RET count (*P* = 0.002), bone marrow cellularity (*P* < 0.001), and sex (*P* = 0.021). Significant independent predictive factors were not found between RCC and ICUS. Detailed data are presented in Table 3 and Figure 9.We utilized independent predictive factors obtained from the multifactorial analysis in this study (such as initial RET count, sex, and degree of bone marrow hyperplasia) in conjunction with domestic and international diagnostic criteria to conduct a multi-stage diagnosis of primary BMFS in its later stages. Initially, at 3 months post-onset, 127 cases of primary BMFS were diagnosed as AA, 23 as RCC, and 17 as ICUS. Ultimately, after more than a year of follow-up, a comprehensive reassessment was conducted based on the clinical course evolution, repeated laboratory tests, bone marrow examinations (including morphology, pathology, and enzyme-linked staining), as well as genetic/cytogenetic analyses. This resulted in 112 cases (67.07%) being reclassified as AA, 34 cases (20.36%) as RCC, and 21 cases (12.57%) as ICUS. During the follow-up period, 15 patients (11.81%) were reclassified; 11 children were re-diagnosed as RCC, and 4 children were re-diagnosed as ICUS. Detailed data are presented in Figure 1B.

**Table 3:** Multivariate logistic regression analysis of main clinical symptoms and laboratory chaaracteristics among AA, RCC and ICUS.

Independent variable	Model 1（AA vs. RCC)					Model 2（AA vs. ICUS)					Model 3 (RCC vs. ICUS)				
	*B*	OR	95%CI		*P*-value	*B*	OR	OR 95%CI		*P*-value	*B*	OR	95%CI		*P*-value
			Lower	Upper				Lower	upper				Lower	Upper	
Absolute reticulocyte count at initial diagnosis	−0.029	0.972	0.954	0.990	0.002	−0.032	0.968	0.945	0.992	0.010	−0.004	0.996	0.966	1.031	0.777
Age	−1.156	0.315	0.118	0.843	0.021	−2.483	0.083	0.018	0.382	0.001	−1.327	0.265	0.057	1.238	0.091
Bone marrow hyperplasia on initial aspiration	1.889	6.615	2.688	16.405	0.000	2.553	12.845	3.703	44.557	0.000	0.664	1.942	0.512	7.382	0.329

In the univariate analysis, factors with statistical significance were selected as independent variables for multivariate analysis using unordered multinomial logistic regression, taking the ICUS group as the control. In logit model 2, comparing AA with ICUS, red blood cell count, the degree of proliferation in bone marrow aspirate smear, and gender were and gender were identified as independent predictors. Specifically, for every increase of 1 × 10^9^/L in red blood cell count, the odds of diagnosing primary BMFS as AA was 0.965 times that of ICUS; compared to those with active proliferation in the bone marrow aspirate smear, those with reduced proliferation had 15.574 times the odds of being diagnosed with AA; and compared to females, males had 0.066 times the odds of being diagnosed with AA. In logit model 3, comparing RCC with ICUS, no independent predictors affecting the diagnosis were found. Using RCC as the control in logit model 1 for comparing AA with RCC, red blood cell count, the degree of proliferation in bone marrow aspirate smear, and gender were identified as independent predictors. Specifically, for every increase of 1 × 10^9^/L in red blood cell count, the odds of diagnosing primary BMFS as AA was 0.974 times that of RCC; compared to those with active proliferation, those with reduced proliferation had 5.857 times the odds of being diagnosed with AA; and males had 0.339 times the odds of being diagnosed with AA compared to females. During the multivariate analysis, abnormalities in bone marrow cell development were found to have an independent predictive effect on the diagnosis of primary BMFS; however, due to an excessive number of cases without developmental abnormalities in AA and an excessive number with abnormalities in RCC, the distribution among the groups was extremely unbalanced, resulting in either excessively large or small odds ratios (ORs). Therefore, this variable was not included in the multivariate analysis.

### Treatment and efficacy

Aplastic Anemia (AA) (*n* = 112): Eleven patients received first-line immunosuppressive therapy (IST) with cyclosporine (CsA), of which 3 achieved complete remission and continued CsA treatment. Ninety-six patients underwent IST but either did not respond to treatment or became transfusion-dependent/severely infected, subsequently receiving hematopoietic stem cell transplantation (HSCT). Five patients were lost to follow-up regarding treatment outcomes.

Refractory Cytopenia in Children (RCC) (*n* = 34): Five patients received CsA treatment (2 of whom responded to CsA and continued treatment), while 3 patients had a brief trial of CsA but were deemed non-responders. Twenty-six patients ultimately underwent HSCT due to IST failure or high-risk characteristics. Three patients were lost to follow-up.

Idoipathic Cytopenia of Uncertain Significance (ICUS) (*n* = 21): Four patients received cyclosporine A treatment (two of whom achieved remission and gradually reduced their dosage). Fourteen patients underwent hematopoietic stem cell transplantation (HSCT) after initial treatment failure or persistent cytopenia. Many ICUS patients (*n* = 21) initially received supportive therapy (such as “saponins, huangbai, and androgens”) before considering the use of immunosuppressants or HSCT. Three patients were lost to follow-up.

Assessment of acute graft-versus-host disease (aGVHD) manifestations within 100 days post-transplantation was performed, categorizing them into mild aGVHD and severe aGVHD. Cases with aGVHD severity ≤II° were included in the mild aGVHD group, while those with aGVHD severity ≥ III°, II° grade 3 skin aGVHD, and II° involving two or more organs were classified as severe aGVHD.

In the RCC group, 20 children experienced mild aGVHD, and 6 experienced severe aGVHD. A comparison of the duration of cyclosporine use prior to transplantation between the RCC mild aGVHD and severe aGVHD groups showed that the mild aGVHD group had a shorter duration of cyclosporine use than the severe group, with statistical significance (*U* = 95.000, *P* = 0.033). In the ICUS group, there were 6 children with mild aGVHD and 7 with severe aGVHD. The duration of cyclosporine use in the mild aGVHD group was also shorter than that in the severe group (*U* = 37.500, *P* = 0.014).

## Discussion

Primary BMFS is a heterogeneous group of disorders caused by acquired factors, characterized by impaired proliferation, differentiation, and maturation of hematopoietic stem or progenitor cells. The most common subtypes include AA, MDS, and ICUS (1–4). AA is a bone marrow failure disorder characterized by reduced nucleated bone marrow cell proliferation and peripheral blood cytopenia affecting two or three lineages (20, 21). RCC is the most prevalent subtype of pediatric MDS, accounting for more than 50% of cases. It is characterized by myeloid dysplasia and ineffective hematopoiesis in the bone marrow, without an increase in blast cells (7, 8, 23, 24). ICUS is defined as persistent cytopenia affecting one or more hematopoietic lineages for at least four months, without meeting the minimal diagnostic criteria for MDS or any other hematologic or systemic disorder (23, 24).

There is currently no definitive consensus on the treatment for ICUS. The diagnosis of ICUS is fundamentally an exclusionary diagnosis aimed at identifying cases with persistent cytopenias that do not meet the clear diagnostic criteria for Aplastic Anemia (AA) or Myelodysplastic Syndromes (MDS), while also excluding other known causes (23, 24). However, the diagnostic criteria for ICUS are primarily based on adult studies, with limited data available for children. The clinical features, natural history, and potential etiologies of ICUS in children (possibly representing a distinct subtype of pediatric idoipathic cytopenia or an early stage of certain diseases) remain to be clarified. This study includes cases that meet the definition of ICUS based on existing standards and long-term follow-up, aiming to explore their early characteristics and provide insights for further understanding of this type of disease.

These disorders initially present with pancytopenia and bone marrow failure, sharing overlapping clinical characteristics, laboratory findings, and histopathological features (4, 9, 23). Their significant heterogeneity and chronic disease course often necessitate long-term follow-up and repeated evaluations by specialists to establish a definitive diagnosis, posing considerable challenges in clinical practice. A study by Forester et al. (26) re-evaluated 72 patients initially diagnosed with AA by three pathologists and found that 10 cases were actually RCC. While AA is a benign disorder, both RCC and ICUS carry a risk of progression to malignant myeloid leukemia (23). In a study by Zhu et al. (9) involving 1,420 cases of NSAA, 152 cases were subsequently reclassified as RCC, with a 10% clonal progression rate. The estimated 5-year and 10-year clonal evolution rates were 15.3% and 20%, respectively. Similarly, Chio et al. (11) followed 61 ICUS patients, of whom one case progressed to MDS. Additionally, Cargo et al. (27) analyzed 4,835 ICUS patients, identifying 82 cases that progressed to MDS or AML. Among the 69 ICUS cases that did not progress, 91% exhibited genetic mutations or chromosomal abnormalities upon monitoring. Given the differences in pathogenesis among BMFS subtypes, their treatment response and risk of disease progression vary considerably. Consequently, early recognition and accurate differential diagnosis at disease onset remain major research challenges in the field.

In summary, the early clinical manifestations of this type of disease are similar, and there is an overlap in clinical and histopathological features, which can lead to misdiagnosis in the early stages, affecting treatment choices and prognosis (11, 23, 26). ICUS are often diagnosed as AA upon presentation and are given IST treatment early in the disease course. Notably, some patients with ICUS do not have a disease mechanism mediated by immune disorders leading to bone marrow damage, resulting in very low efficacy of IST (11, 23, 24). These patients may experience a protracted disease course with a high risk of severe and recurrent infections, as well as the potential for clonal evolution, leading to progressively worsening peripheral blood counts. The history of long-term medication use, recurrent infections, worsening blood counts, and family anxiety contribute to a strong desire for transplantation among this population. In some cases, patients may undergo hematopoietic stem cell transplantation (HSCT), which carries a transplant-related mortality risk that far exceeds the risks associated with the disease itself (20, 21, 24, 28). Furthermore, some children with ICUS may have an indolent disease course that could be self-limiting and persist for many years without progression. This portion of the population does not require treatment but rather long-term monitoring of blood counts (7, 24, 28).

Therefore, the early misdiagnosis of ICUS as AA may lead to inappropriate treatment with immunosuppressive agents, resulting in ineffective medication and exacerbation of the condition. For instance, among the 21 ICUS patients in our study, 19 had received CsA treatment at outside institutions based on a presumed diagnosis of AA prior to referral to our center, but the response rate was extremely low (only about 10.5%). If patients with severe Aplastic Anemia (SAA/VSAA) or high-risk RCC are initially misdiagnosed with a milder condition (such as Non-Severe AA (NSAA) or ICUS), this may delay the initiation of timely and necessary intensified treatments, such as immunosuppressive therapy (IST) or early hematopoietic stem cell transplantation (HSCT) (20, 21, 26). Furthermore, we observed that the longer the duration of CsA treatment before transplantation in the RCC group, the higher the risk of developing severe acute graft-versus-host disease (GVHD) post-transplant. The above content clearly demonstrates that the uncertainty in early diagnosis significantly affects the diagnostic pathways and early treatment, leading to issues such as ineffective treatment, treatment delays, and an increased risk of clonal progression. Therefore, it is crucial to predict the diagnosis based on clinical and laboratory characteristics in the early stages of the disease.

Our retrospective analysis revealed that AA presents with more severe clinical manifestations at disease onset compared to RCC and ICUS, progresses more rapidly, and is more likely to result in transfusion dependence and recurrent infections. This study examined the initial blood cell counts at disease onset and the lowest blood cell values within the first three months of disease onset. The findings demonstrated that initial PLT and ANC counts in AA were significantly lower than those in RCC and ICUS, and the lowest PLT and ANC values within three months were also markedly lower in the AA group, indicating that bone marrow failure is most severe in AA, while RCC and ICUS exhibit relatively milder hematopoietic dysfunction (11, 29–33). These results suggest that in newly diagnosed primary BMFS, cases characterized by markedly abnormal peripheral blood counts at presentation and a rapid decline within the first three months are more likely to be AA.

This study also systematically compares erythrocyte parameters among patients withAA, RCC and ICUS. MCV reflects the average red blood cell size, while RDW assesses size variability, with elevated RDW indicating ineffective erythropoiesis or abnormal morphology. MCH quantifies the average hemoglobin content per red blood cell (34–36). Our results showed that patients with AA had significantly lower MCV, MCH, and RDW compared to those with RCC, characterized by clonal hematopoiesis and abnormal erythroid development, leads to heterogeneous red blood cell sizes and increased RDW. In contrast, AA is associated with a profound depletion of bone marrow hematopoietic stem cells, resulting in a more uniform red blood cell population with lower RDW (20, 21). Traditionally, RCC has been ssociated with macrocytic anemia (MCV > 100 fL) and AA with normocytic anemia (MCV 80–100 fL) (34). However, our study revealed that 60% of RCC cases fell within the normocytic range, while 19.3% of AA patients with macrocytosis. This finding underscores the limitations of using MCV alone for differential diagnosis. Therefore, a combination of MCV, RDW, and other erythrocyte parameters is recommended for early diagnosis (34, 37, 38). Specifically, in patients with primary BMFS, a low MCV and RDW suggest AA, whereas a normal or elevated MCV with high RDW is more indicative of RCC or early clonal hematopoiesis.

RET count serves as an indicator of bone marrow hematopoietic potential. A 2015 collaborative study conducted by the Chinese Academy of Medical Sciences Tianjin Institute of Hematology and Japan analyzed 100 patients and found that RET counts in AA patients were significantly lower than those in RCC patients (6). Consistently, our study also demonstrated that RET count and percentage were significantly lower in AA patients compared to RCC and ICUS. The underlying mechanism is likely related to bone marrow replacement by adipocytes in AA, which severely impairs erythroid hematopoiesis. In contrast, while RCC is characterized by dysplastic hematopoiesis, erythropoiesis remains relatively active, leading to higher RET counts and percentages compared to AA. However, due to ineffective hematopoiesis, peripheral blood hemoglobin levels remain low in RCC. ICUS hematopoietic function appears to be intermediate between AA and RCC, reflecting a less severe but persistent impairment of bone marrow function.

Bone marrow examination is a crucial diagnostic tool for bone marrow failure syndromes (BMFS) (29). However, morphological assessment is inherently subjective and may be influenced by disease progression and specific sampling sites. In our study, 22.6% of AA patients initially exhibited hypercellular bone marrow on smear, which may be associated with chronic AA or sampling from specific sites such as the sternum or spinous processes during the early disease stage. Therefore, multisite bone marrow aspiration is recommended for BMFS diagnosis, along with repeated evaluations during follow-up. Additionally, bone marrow dysplasia is a key diagnostic feature of MDS, but studies have reported that AA can also exhibit dysplastic hematopoiesis similar to MDS, which may be attributed to folate or vitamin B12 deficiency, early immunosuppressive therapy, or severe bone marrow failure (29, 31, 33). However, the precise mechanisms remain to be further investigated. Our study found that bone marrow dysplasia was significantly more pronounced in RCC, with higher proportions of single-lineage and bilineage abnormalities in RCC compared to AA and ICUS. Regarding micromegakaryocyte enzyme labeling, RCC exhibited the most diverse abnormalities, including large mononuclear micro-MK, binucleated megakaryocytes, and multinucleated megakaryocytes, whereas abnormal findings in AA and ICUS were relatively limited and less diverse.

These characteristic differences arise from the distinct pathogenesis of different BMFS subtypes. AA is primarily driven by immune dysregulation, which directly damages hematopoietic stem and progenitor cells (HSCs/HPCs). In contrast, RCC results from somatic mutations in mature hematopoietic stem cells, which drive clonal hematopoiesis, making stem/progenitor cell differentiation and maturation defects the primary pathogenic mechanism. The pathogenesis of ICUS remains unclear, but it is hypothesized to involve multiple contributing factors, including abnormalities in the hematopoietic microenvironment and mild immune dysregulation (23, 24). As a result, AA manifests with severe peripheral cytopenia early in the disease course, whereas ICUS and MDS exhibit a slower progression with relatively milder disease severity. Additionally, RCC is more prone to the emergence of complex dysplastic bone marrow cells compared to the other two subtypes.

Furthermore, multivariate analysis identified initial RET count, sex, and bone marrow cellularity as independent predictive factors for the diagnosis of AA in primary BMFS. Specifically, a lower initial RET count, hypocellular bone marrow on the first smear, and female sex were associated with a higher likelihood of AA diagnosis. In contrast, a higher initial RET count, hypercellular bone marrow, and male sex were more indicative of ICUS or RCC. However, no significant independent predictors were identified to distinguish between RCC and ICUS. These findings provide new insights for the early differential diagnosis of pediatric BMFS.

The uniqueness of this study lies in the simultaneous comparison of the early characteristics (within the first three months) of the three easily confusable diseases: AA, RCC, and ICUS, within a pediatric cohort. Through multifactorial analysis, we identified statistically significant independent predictive factors (such as initial RET count, sex, and degree of bone marrow hyperplasia), which provide new objective evidence for clinical differentiation during the diagnostic ambiguity period. Our study found that 16 patients who were ultimately diagnosed with ICUS received IST because they were initially diagnosed with AA. Additionally, 18 children with RCC were diagnosed with AA at an early stage, but high-risk RCC patients did not receive timely transplant treatment. If we utilized independent predictive factors obtained from the multifactorial analysis in this study (such as initial RET count, sex, and degree of bone marrow hyperplasia) in conjunction with domestic and international diagnostic criteria to conduct a multi-stage diagnosis of primary BMFS in its later stages, only 4 ICUS patients would have been diagnosed with AA early, potentially avoiding unnecessary immunosuppressive therapy in 14 patients. Similarly, only 11 RCC patients would have been diagnosed with AA early, allowing for timely and necessary intensive treatment. Detailed data are presented in Figure 1B. In summary, the reclassified data underscore the clinical necessity of addressing this issue. This study represents a significant step forward in tackling this problem by enabling the prediction and diagnosis of the disease at an early stage based on clinical and laboratory characteristics. This approach aims to implement more precise treatments to enhance efficacy.

This study has certain limitations, including a relatively small sample size and a single-center design focusing on pediatric patients from a specific region, which may introduce regional bias. Future research should aim to expand the sample size and conduct multi-center, large-scale prospective studies to enhance the accuracy and generalizability of the findings. Additionally, the follow-up period in this study was relatively short, and long-term treatment outcomes and prognosis were not comprehensively analyzed. Future studies should focus on long-term clinical outcomes, investigating the impact of early diagnosis and intervention on prognosis. Furthermore, additional molecular biomarkers and biological characteristics need to be explored (27, 36, 39). Integrating these markers into a comprehensive early diagnostic and predictive model could facilitate early identification, risk stratification, and personalized treatment, ultimately improving patient outcomes.

## Data Availability

The original contributions presented in the study are included in the article/Supplementary Material, further inquiries can be directed to the corresponding author.
